# **Outcomes of transmediastinal esophagectomy for patients with esophagogastric junction cancers**

**DOI:** 10.1007/s10147-026-03068-1

**Published:** 2026-05-27

**Authors:** Kotaro Sugawara, Koichi Yagi, Shoh Yajima, Yoshiyuki Miwa, Shuichiro Oya, Asami Okamoto, Haruki Kojima, Raito Asaoka, Yoshifumi Baba

**Affiliations:** Department of Gastrointestinal Surgery, Graduate School of Medicine, The University of Tokyo, 7-3-1 Hongo, Bunkyo-ku, Tokyo, 113-8655 Japan

**Keywords:** Esophagogastric junction cancer, Transmediastinal esophagectomy, Anastomotic leakage

## Abstract

**Purpose:**

We aimed to evaluate the outcomes of transmediastinal esophagectomy (TME) for esophagogastric junction cancer (EGJC) patients.

**Methods:**

We retrospectively reviewed patients who underwent TME for EGJC (Siewert Type II) between 2018 and 2023. Robot-assisted TME was performed using a robotic surgical system (DaVinci). Postoperative complications, overall survival (OS) and disease-free survival (DFS) were investigated.

**Results:**

The median length of esophageal invasion was 3 cm. Adenocarcinoma was the most common tumor in our series (71.1%). A robotic approach was used for 25 (65.8%) patients. The most frequent major complication was pneumonia (≥ Grade I, *n* = 12, 31.6%), followed by recurrent laryngeal nerve palsy (≥ Grade I, *n* = 6, 15.8%). The incidence of anastomotic leakage (≥ Grade II) was 5.3% (*n* = 2). The 30-day mortality rate was 0%. The 3-year OS and DFS rates were 67.5 and 51.8%, respectively. The distribution of pathological LN metastases did not significantly demarcate OS curves (*P* = 0.43), while significantly stratifying DFS curves (*P* < 0.001). Patients with both mediastinal and abdominal LN metastases had very poor DFS (2-year DFS; 0%).

**Conclusions:**

TME for EGJC can be performed safely without increasing complications such as severe anastomotic leakage and in-hospital death. The relatively high frequency of pneumonia should be considered.

**Supplementary Information:**

The online version contains supplementary material available at 10.1007/s10147-026-03068-1.

## Introduction

Esophageal carcinoma is still a major cause of cancer-related mortality worldwide, despite the recent advances in diagnosis and multimodal treatments [1]. Notably, the prevalence of esophagogastric junction cancer (EGJC) has been rising in both Eastern Asia and Western countries [2]. Although a recent Japanese prospective nationwide multicenter study suggested an optimal extent of subsequent lymph node (LN) dissection [3], the surgical approach has yet to be standardized for EGJC [4].

For technical reasons, the short-term outcomes after surgery for EGJC remain unsatisfactory [3]. In particular, postoperative anastomotic leakage, which adversely affects clinical outcomes, has been reported in approximately 11–13% of patients who underwent surgery for EGJC [3, 5]. Recent studies revealed intrathoracic anastomotic leakage to readily induce mediastinitis and empyema, thereby leading to severe systemic infectious disease, resulting in prolonged hospitalization and poor long-term oncological outcomes [6, 5].

Cervical anastomosis with subtotal esophagectomy may thus be an option for EGJC patients [6]. Given that conventional transthoracic subtotal esophagectomy reportedly reduces cardiopulmonary functions and also quality of life, as compared with a transabdominal approach [7, 8], minimally invasive esophagectomy has attracted the attention of surgeons as a potentially less invasive alternative [9, 10].

We recently developed “robot-assisted transmediastinal esophagectomy (TME)” and have demonstrated that this approach can be safely performed while retaining oncological radicality [11, 12]. Robot-assisted TME features a combination of transcervical mediastinoscopy, transhiatal laparoscopy and transhiatal robotic procedures [13, 14]. Neither unilateral lung ventilation nor a transthoracic approach are required to carry out the TME procedure. Therefore, TME can be performed safely even in cases with low pulmonary function and/or severe intrathoracic adhesions.

The outcomes of patients who underwent TME for EGJC have not, to our knowledge, been investigated previously. Herein, we studied the short- and long-term outcomes of patients who underwent surgery employing our new approach.

## Patients and methods

### Patients

The following eligibility criteria were applied: (1) patients who underwent surgery between May 2018 and April 2023 for EGJC (tumor epicenter located within 2.0 cm of the EGJ) [15]; (2) histologically-confirmed adenocarcinoma (AC) or squamous cell carcinoma (SCC); (3) tumor deemed to be resectable; (4) one-staged gastric tube reconstruction. Prior to mediastinoscopic esophagectomy being approved by the National Health Insurance system in April 2018, robot-assisted TME was performed as part of an independent clinical trial.

During the study period, 300 patients underwent curative resection for esophageal cancer. Among them, 249 had thoracic esophageal cancer (including Siewert type I) and 12 had cervical esophageal cancer. Of the remaining 39 patients with esophagogastric junction cancer (Siewert type II), one patient who underwent staged reconstruction after prior gastrectomy was excluded. The final study cohort comprised 38 patients.

The patients’ clinical records, prospectively maintained in a database, were retrospectively reviewed. At the time of the final follow-up (May 2025), the median follow-up period was 42.9 (range, 6.1 to 79.5) months for the survivors. This retrospective study was approved by the local ethics committee of the faculty of medicine at the University of Tokyo (ID: 3962).

### Treatment strategy

Clinical and histological tumor staging was based on the TNM classification (UICC, 8th edition) [16]. The choice of treatment strategy was discussed at a biweekly multidisciplinary cancer board meeting attended by radiologists, gastroenterologists and surgeons, with decisions being made in accordance with the Guidelines for the Diagnosis and Treatment of Carcinoma of the Esophagus [17]. Neoadjuvant chemotherapy (NAC) was administered to cT2-3 N+ patients. For cT1N1, cT2N0 and a portion of the cT3N0 patients, NAC was not given. The standard NAC regimen was three cycles of DCF (docetaxel, 70 mg/m2 on day 1; cisplatin, 70 mg/m2 on day 1; 5-FU, 700 mg/m2 on day 1–5) every 3 weeks. Postoperative chemotherapy was administered to patients with pN + disease stage.

At our institution, LN dissection for esophagogastric junction cancer is performed according to the following criteria: (1) When esophageal invasion exceeds 2 cm, lower and middle mediastinal LN dissection is performed. (2) When esophageal invasion exceeds 3 cm, lower, middle, and upper mediastinal LN dissection is performed. (3) Even when esophageal invasion is 1–3 cm, lower, middle, and upper mediastinal LN dissection is performed in cases with cN + in the upper/middle mediastinum or with SCC histology. Because the transhiatal robotic approach enables safer and more precise middle mediastinal LN dissection [18], and to avoid a high intramediastinal anastomosis, TME is generally performed even in cases meeting criterion 1.

### Surgical treatment and postoperative management

Robot-assisted transmediastinal esophagectomy, or TME, with two- or three-field lymphadenectomy was performed using a robotic surgical system, da Vinci S or Xi (Intuitive Surgical, Sunnyvale, CA, USA), as described in previous publications [19, 20]. For Siewert type II tumors, two-field lymphadenectomy was generally performed. In our practice, three-field lymph node dissection was performed in patients without significant surgical risk when upper mediastinal lymph node nodal involvement was suspected.

Briefly, all surgical procedures were performed with the patient in the supine position, with bilateral lung ventilation (Supplementary Fig. 1). The cervical and right upper mediastinal LNs were dissected employing a cervical incision. Since 2015, left upper mediastinal LN dissection has been performed using a mediastinoscopic procedure under pneumo-mediastinum conditions. During the study period, intraoperative neuromonitoring was not utilized during the mediastinoscopic procedure. Abdominal and lower mediastinal LN dissections were performed using a laparoscopic procedure, simultaneously with the cervical procedure. Remnant lower and middle mediastinal LN dissections, including subcarinal and main bronchial LN dissections, were performed employing a transhiatal robotic (DaVinci) procedure. We performed hand-sewn anastomosis at the level of the cervix using a gastric conduit via the posterior mediastinal route for all patients included for the study.

### Definition of complications

Postoperative complications were defined as adverse events occurring within 30 days of surgery or during the in-hospital period, and their severities were assessed using the Clavien–Dindo (C–D) classification [21]. Each complication was categorized according to the international consensus [22]. We focused mainly on pulmonary complications, clinically or radiologically proven anastomotic leakage and recurrent laryngeal nerve palsy (RLNP).

Pulmonary complications were defined as the presence of one or more of the following postoperative conditions [23, 24]: intubation for respiratory failure, acute respiratory distress syndrome (ARDS), pneumonia and atelectasis requiring bronchoscopy or antibiotics. Pneumonia was defined as new lung infiltrates on chest radiography with clinical evidence of an infectious origin, including newly onset fever, purulent sputum, leukocytosis, and decline in oxygenation [25].

Anastomotic leakage was defined as clinical signs of leakage, such as erythema, skin edema, emission of saliva or pus from a surgical wound or cervical drain or a radiographically apparent leak confirmed by performing esophagography or computed tomography or both [26].

Attending surgeons and anesthesiologists routinely assessed vocal cord movement via immediate postoperative bronchoscopic observation after extubation [18]. Also, we assessed the severity of RLNP based on symptoms that occurred and resolved during hospitalization, such as voice hoarseness and dysphagia. If only hoarseness or dysphagia was observed and treatment was not required, the patient’s condition was classified as C-D grade I.

### Statistical analysis

Categorical variables were expressed in numerical figures and percentages and compared using Fisher’s exact test or the χ2 test, as appropriate. Continuous variables were expressed as the median values (range) and compared using Wilcoxon’s rank-sum test. Statistical analyses were carried out using JMP Student Edition 18.0.0 (SAS Institute, Cary, NC).

## Results

### Patient characteristics

Table 1 lists the characteristics of our patients. The median age was 69 years, and most patients were male (86.8%). The median length of esophageal invasion was 3 cm (range, 1–5 cm). Adenocarcinoma was the most common tumor in our series (71.1%). Our cohort included 4/9/17/8 patients with cStage I/II/III/IV, respectively. Thirty-one (81.5%) patients had advanced (pStage III-IV) esophageal cancer. The median numbers of harvested total LNs, mediastinal LNs and abdominal LNs were 46 (range, 27–89), 25 (2–56) and 22 (11–44), respectively.

**Table 1:** Characteristics of 38 patients with esophagogastric junctional cancers

Variables	No. of patients (%)
Age, y Median (range)	69 (38–82)
Sex, male; female	33 (86.8)/ 5 (13.2)
BMI, Median (range)	24.2 (16.9–26.7)
Siewert Type I/II	0 (-)/ 38 (100)
Esophageal invasion, cm Median (range)	3 (1–5)
Histology, SCC/AC	11 (28.9)/ 27 (71.1)
cStage, I/II/III/IV	4 (10.5)/ 9 (23.7)/ 17 (44.7)/ 8 (21.1)
Neoadjuvant (Induction) therapy	20 (52.6)
DCF	11 (28.9)
pStage, I/II/III/IV	1 (2.7)
CF	4 (10.5)
XELOX	2 (5.3)
FOLFOX	2 (5.3)
SOX+Nivo	5 (13.2)/ 2 (5.3)/ 22 (57.9)/ 9 (23.6)
The number of harvested LNs, Median (range)	46 (27–89)
Mediastinal LNs, Median (range)	25 (2–56)
Abdominal LNs, Median (range)	22 (11–44)
The number of metastatic LNs, Median (range)	2 (0–18)

*BMI*, body mass index; *SCC*, squamous cell carcinoma; *AC*, adenocarcinoma

## Surgical outcomes

Surgical outcomes are shown in Table 2. All patients underwent R0 resection (all surgical margins (proximal, distal, and circumferential) were negative.). A minimally-invasive abdominal approach was employed in most patients (94.7%). A robotic transabdominal approach was used for 25 (65.8%) patients. Two- or three-field lymphadenectomy was performed for 33 (86.8%) and 5 (13.2%) patients, respectively. The median operation time was 423 min (range, 289–593 min).

**Table 2:** Surgical outcomes of 38 patients

Variables	No. of patients (%)
Abdominal approach, laparo/open	36 (94.7)/ 2 (5.3)
Use of robot	25 (65.8)
LND, 2-field/3-field	33 (86.8)/ 5 (13.2)
Operation time, Min Median (range)	423 (289–593)
Blood loss, ml Median (range)	160 (10–755)
*Postoperative complications*	
Overall, Grade II/III/IV/V	16 (42.1)/ 5 (13.2)/ 1 (2.6)/ 0 (-)
Pneumonia, Grade II/III	11 (29.0)/ 1 (2.6)
Anastomotic leakage (≥ Grade II)	2 (5.3)
Recurrent laryngeal nerve palsy (≥ Grade I)	6 (15.8)
Hospital stay	18 (11–117)

*LND*, lymph node dissection

The overall morbidity rate was 58.1% (22/38). The most common major complication was pneumonia (≥ Grade I, *n* = 12, 31.6%), followed by RLNP (≥ Grade I, *n* = 6, 15.8%). The incidence of anastomotic leakage (≥ Grade II) was 5.3% (*n* = 2). The 30-day mortality rate was 0%. One patient (2.6%) developed severe complications (≥ Grade IIIb). Patients who developed pneumonia had a significantly higher incidence of RLNP (4/12, 33.3%) than those who did not (2/26, 7.7%) (*P* = 0.04).

Operative time, blood loss, incidence of postoperative complications (anastomotic leakage and RLNP), and length of hospital stay did not differ significantly between the early (May 2018–August 2021) and late (September 2021–April 2023) periods of the study.

## The dissection and metastatic rates of LNs

**Fig. 1:**
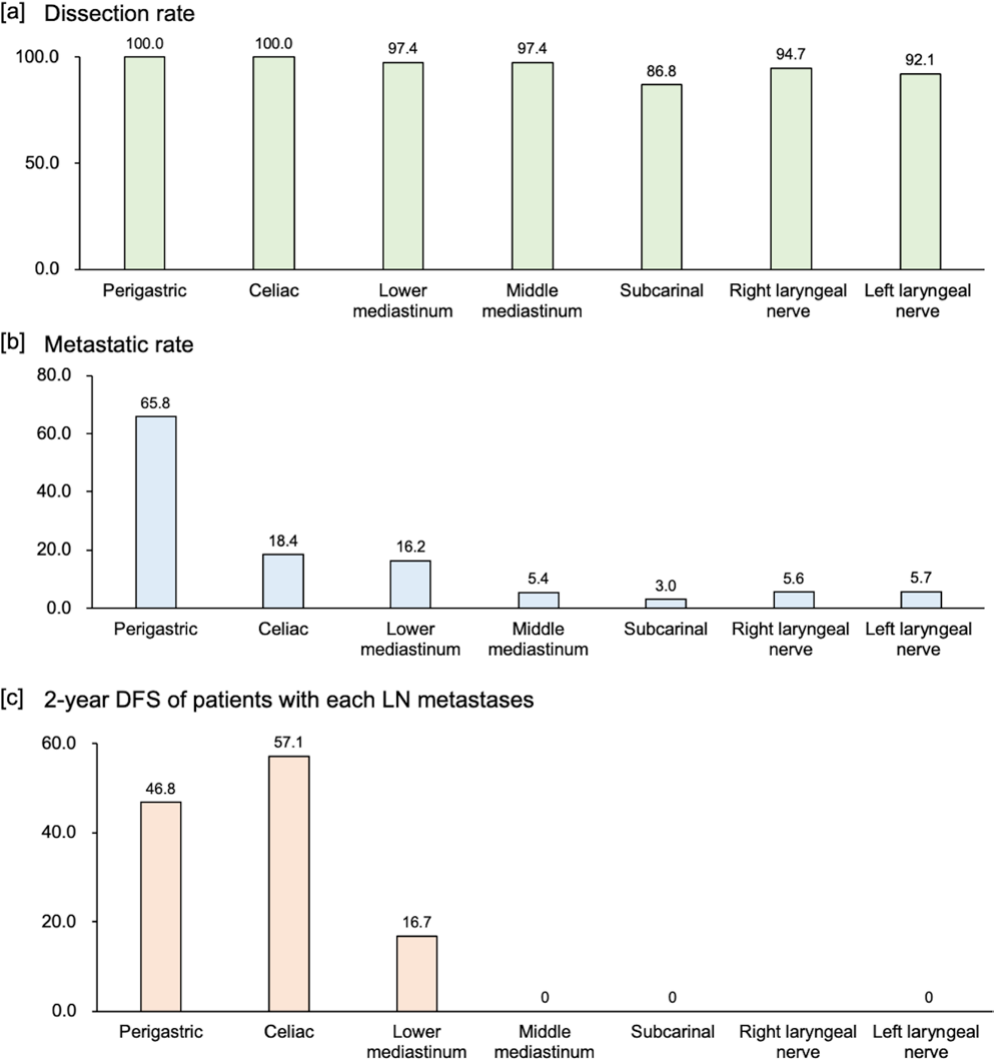
Pathological findings and survival impacts of nodal involvement at each LN station. **a** Dissection and **b** metastatic rates for each LN station in 38 patients with EGJC. **c** 2-year DFS of patients with metastasis at each LN station

Next, we studied the dissection and metastatic rates of LNs in our series. Supplementary Table 1 outlines the node zone names used in our study. Figure 1a illustrates the dissection rate of regional LNs. Abdominal LNs were harvested in all cases. Subcarinal LN dissection was omitted in five cases, laryngeal nerve in three. Metastatic LNs were mainly distributed among the abdominal (perigastric and celiac) and lower mediastinal LNs (Fig. 1b). The incidence of middle mediastinal, subcarinal and laryngeal nerve LN metastases was approximately 5% (Fig. 1b). The extent of esophageal invasion, histology and cT category did not differ significantly between patients with upper or middle mediastinum LN metastases (*n* = 5) and those without (*n* = 33) (Supplementary Table 2).

Patients with abdominal LN metastases had relatively good DFS, while those with mediastinal LN metastases, especially metastatic disease involving upper/middle mediastinal nodes, had very poor DFS (Fig. 1c).

### Survival outcomes

**Fig. 2:**
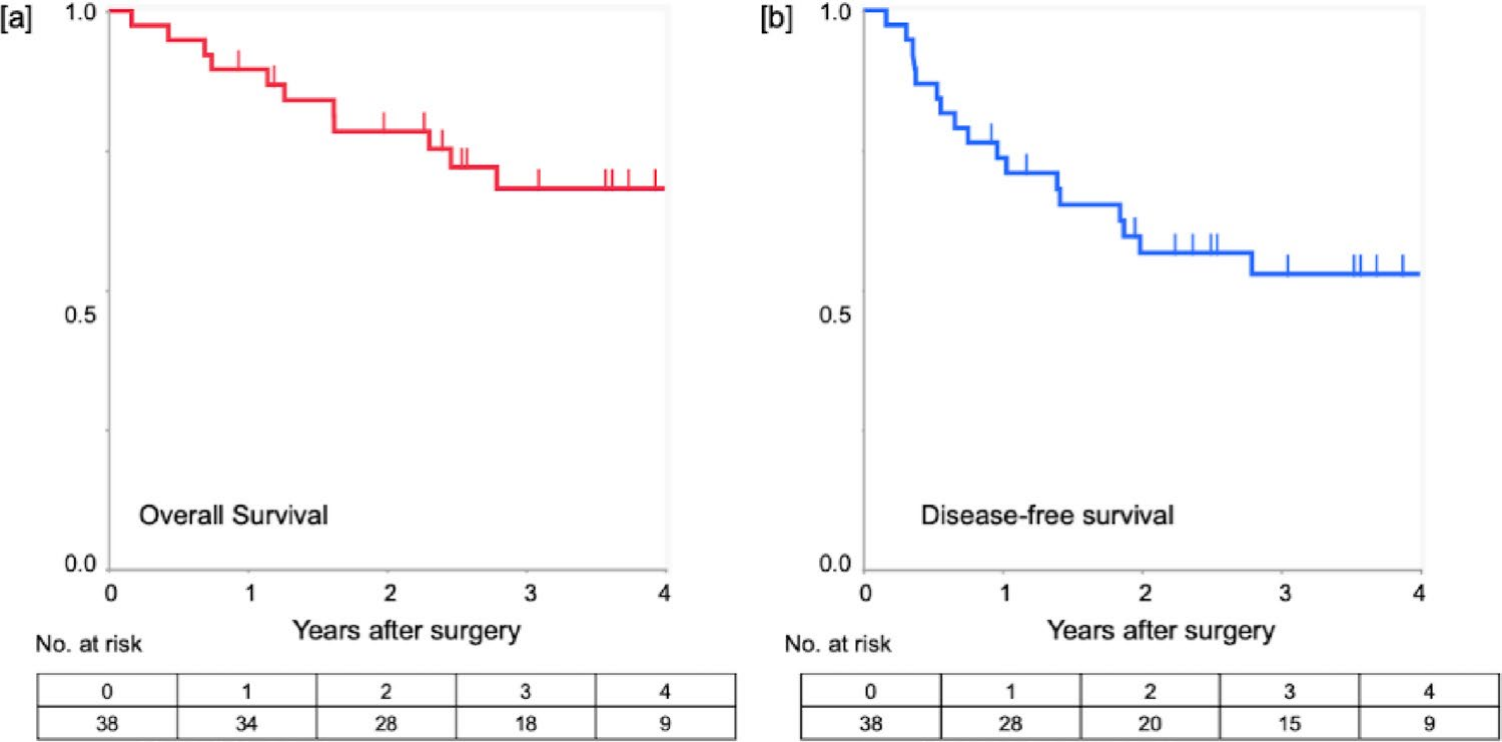
Survival outcomes of our series. **a** Overall survival and **b** disease-free survival curves of 38 patients

The 3-year OS and the DFS rates of our series were 67.5% and 51.8%, respectively (Fig. 2). The OS and DFS curves were not significantly demarcated according to tumor histology (*P* = 0.42 and *P* = 0.28, respectively, Supplementary Fig. 2).

**Fig. 3:**
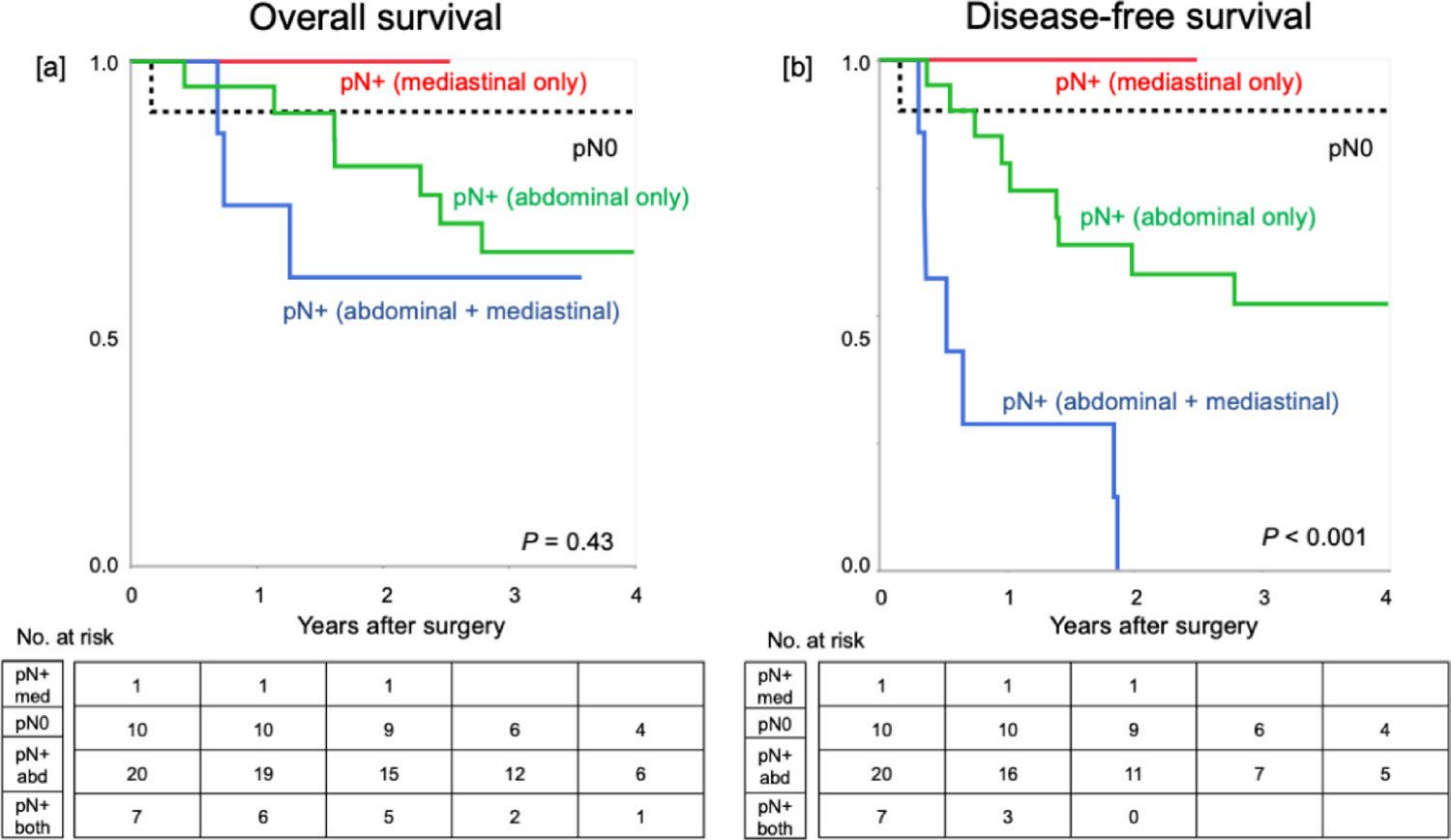
Survival outcomes according to pathological LN metastasis. **a** Overall survival and **b** disease-free survival curves according to the distribution of pathological LN metastasis. **a** Patients who had both mediastinal and abdominal LN metastasis had very poor DFS. **b** OS was not significantly demarcated by the distribution of pathological LN metastasis

OS curves were not significantly demarcated by the distribution of pathological LN metastases (*P* = 0.43, Fig. 3a). One patient without pN+ died due to aortic dissection 2 months after surgery. In contrast, the DFS curves were significantly stratified by the distribution of pathological LN metastases (*P* < 0.01, Fig. 3b). Significant survival differences were observed irrespective of tumor histology, although the small SCC subcohort should be noted (Supplementary Fig. 3). Patients with both mediastinal and abdominal LN metastases had very poor DFS (2-year DFS; 0%). One patient with only mediastinal LN metastases survived more than 2 years without recurrent disease.

Patients with both abdominal and mediastinal LN involvement had significantly higher number of metastatic LNs (median; 11, range 4–18) than those with abdominal or mediastinal LN involvement alone (median; 2, range 1–13). Supplementary Table 3 summarizes the recurrence pattern observed in our series. Among 16 patients who had recurrences, only one developed a locoregional recurrence alone (upper mediastinal LN). Systemic recurrence and mixed recurrence were seen in 12 and 3 patients, respectively. Among patients with both abdominal and mediastinal LN metastases, all recurrences were distant (7/7). In contrast, distant metastases were absent in 4 of 9 patients (44%) with either abdominal or mediastinal LN metastasis (Supplementary Fig. 4).

## Discussion

We investigated surgical outcomes, LN involvement and survival after TME. The majority of our study subjects had adenocarcinoma of EGJC. To our knowledge, this is the first investigation of the outcomes of patients who underwent TME for EGJC.

For Siewert type I-II EGJC, the optimal surgical approach remains a matter of considerable debate. A transthoracic approach ensures adequate proximal margins and allows systematic mediastinal lymphadenectomy [7, 27]. In contrast, while a transhiatal approach avoids thoracotomy, which reduces the risk of respiratory complications and facilitates recovery, its major limitation is the inability to achieve an extensive mediastinal dissection, thereby raising concerns regarding oncological radicality [7, 27]. Importantly, anastomotic leakage, especially when potentially fatal (≥ Grade IV), is reportedly more frequent in patients who underwent surgery via the transhiatal approach for EGJC than in those who had the procedure via a transthoracic approach [5, 6].

While these observations highlight the clinical utility of the transthoracic approach for EGJC, the transthoracic approach can be difficult to perform in patients with impaired pulmonary function or intrathoracic adhesions after lung surgery. The transmediastinal approach overcomes the limitations of other minimally invasive techniques by providing direct access to the upper mediastinum, with adequate LN dissection while preserving the important adjacent anatomical structures [28, 11–14]. A prospective study evaluating the integrity of mediastinal LN dissection with TME suggested mediastinal LN dissection to be adequate with TME alone [29]. In the present study, we first investigated the clinical utility of this novel approach and determined the survival outcomes of patients who underwent TME for EGJC.

In our series, there were no perioperative mortalities, indicating that TME can be safely employed in the patient population with EGJC. The incidence of severe complications (Clavien–Dindo ≥ IIIb) was very low (2.6%), while in a European multicenter study, severe complications (Clavien–Dindo ≥ IIIb) were observed in 17.6% of patients who underwent transthoracic esophagectomy for Type II EGJC [27]. Notably, the incidence of anastomotic leakage (≥ Grade II) was 5.3% in our study. A prospective multicenter study showed anastomotic leakage to occur in approximately 12% of patients operated on via transthoracic or transhiatal approaches, i.e., there was no difference between the two [5]. Taking these results together, TME is deemed to be useful for preventing severe inflammation and mediastinitis caused by leakage from an intrathoracic anastomosis [6].

The high incidences of RLNP (≥ Grade I, 15.8%) and pneumonia (≥ Grade I, 32.6%) in our series merit careful consideration. The incidence of RLNP after TME reportedly ranges from 12 to 31.4% [30–32], thus remaining a significant concern [28, 33]. RLNP was assessed only at the time of extubation, enabling standardized assessment across all patients prior to the initiation of oral intake and avoiding variability related to clinical scheduling. No scheduled reassessment of RLNP was incorporated into the study design. Although postoperative RLNP often resolves within approximately six months in clinical practice, the lack of longitudinal evaluation prevented distinction between transient and permanent palsy in this study. TME requires implementing strategies such as intraoperative neuromonitoring and refined surgical techniques [34]. RLNP was not distinguished as transient or permanent, and assessment was limited to the early postoperative period. In contrast, a recent randomized clinical trial classified RLN palsy by laterality (unilateral vs. bilateral) and duration (temporary, recovering within 6 months, vs. permanent, not recovering within 6 months) [35], representing a major limitation of our study.

In general, TME reportedly reduces the risk of respiratory complications [36, 32]. There are several possible explanations for the high incidence of postoperative pneumonia in our cohort. Because postoperative pneumonia after esophagectomy is common and severe pneumonia is a major cause of postoperative mortality [37], antibiotics were administered at the discretion of the attending physicians when pneumonia was suspected, which likely increased the incidence of grade ≥ 2 pneumonia. Given the limited diagnostic accuracy and potential for overdiagnosis of postoperative pneumonia after esophagectomy, more precise management is required to ensure appropriate antibiotic use [38, 39]. Among the 12 patients with postoperative pneumonia, 11 improved with antibiotic therapy and physiotherapy, and only one required re-intubation due to aspiration pneumonia; severe cases were rare in our series.

It is noteworthy that patients who developed pneumonia had a significantly higher incidence of RLNP than those who did not, a finding that suggests RLNP to be a cause of pneumonia. Considering that LN metastasis along the RLN, especially the left RLN, is reportedly rare in EGJC without extensive esophageal invasion [3], lymphadenectomy along the RLN can be omitted in patients with esophageal invasion < 4 cm and who are negative for clinical LN metastasis [40]. Omitting lymphadenectomy along the RLN might reduce the incidence of pneumonia.

Survival outcomes for our series were acceptable, with 3-year OS and DFS rates of 67.5 and 51.8%, respectively. Notably, OS curves were not significantly influenced by histological subtype, postoperative complications, or LN distribution, whereas DFS curves were clearly demarcated by patterns of LN involvement. This discrepancy highlights the need for careful interpretation of survival metrics, as OS might be impacted by competing risks and subsequent therapies, while DFS more directly reflects the effects of nodal disease. Furthermore, DFS is not a fully validated surrogate for OS, and its interpretation requires caution owing to potential inaccuracies in cause-of-death attribution and the influence of competing risks [41]. Given the small sample size of our series (*n* = 38) and the short follow-up period of our cohort (median; 42.9 months), the survival significance of LN distribution was not fully addressed in our study. The reason for the discrepancy between the impact of the distribution of pathological lymph node metastases on DFS and OS remains unclear. Changes in treatments for recurrent esophageal cancer during the study period (2018–2023), particularly the introduction of immune checkpoint inhibitors, may have contributed to this difference.

Our analysis of LN dissection and metastasis highlighted the prognostic relevance of the metastatic distribution. Abdominal and lower mediastinal LNs were the most common sites of involvement, whereas middle mediastinal, subcarinal, and recurrent laryngeal nerve LNs were rarely affected. Importantly, patients with mediastinal LN metastases, particularly in the upper and middle mediastinum, had dismal DFS, while those with isolated abdominal LN metastases experienced comparatively favorable outcomes. These findings suggest that the biological behavior of mediastinal nodal disease differs from that of abdominal disease and may warrant surgical or adjuvant strategies tailored to individual patients. The survival impacts of nodal distribution might be attributable to the nodal burden in our series. Three-field lymph node dissection was performed when upper mediastinal lymph node metastasis was suspected, but its benefit remains unclear, and results from an ongoing trial in Japan are awaited [42].

A recent Japanese nationwide study accurately identified the distribution of LN metastases from EGJC and the optimal extent of subsequent LN dissection [3]; subtotal esophagectomy with upper mediastinal LN dissection is required for EGJC with esophageal involvement exceeding 4.0 cm. Recurrence is reportedly detected in mediastinal LN in 2.2–5.8% of patients after surgery for Siewert type II tumors [6, 43, 44]. In our series, only 1 patient (2.6%) developed mediastinal recurrence, with no other recurrent lesions, possibly suggesting the oncological adequacy of our TME approach. Taken together, these observations suggest that our TME approach, which is associated with a low incidence of severe complications, is clinically useful for EGJC with extensive esophageal involvement.

Notably, a recent nationwide multicenter prospective study further clarified the optimal extent of LN dissection in esophagogastric junction cancer [45]. Lymphadenectomy should be tailored to the length of esophageal involvement, with extensive mediastinal dissection reserved for patients with long EI, cN+ disease in the upper or middle mediastinum, or those who did not receive neoadjuvant chemotherapy. Although our indications for TME are generally consistent with these criteria, they are somewhat broader and may be overly invasive in some cases. Therefore, optimization of TME indications based on accurate preoperative assessment and a clear treatment algorithm is warranted.

The present study has several limitations. The sample size was relatively small, and the retrospective design may have introduced selection bias. Additionally, variations in surgical approaches and the extent of lymphadenectomy might have influenced nodal yield and metastasis detection. Furthermore, because TME has been broadly applied at our institution, few patients underwent lower esophagectomy with proximal gastrectomy and intramediastinal (especially high) anastomosis during the same period. Consequently, no appropriate control group was available, precluding comparative analysis of short-term outcomes and representing a major limitation of this study. Despite these limitations, our study provides novel insights into treatment strategies for EGJC.

In summary, TME is potentially feasible for EGJC, given that none of our patients experienced severe anastomotic leakage or in-hospital death. Still, the relatively high frequency of RLNP and pneumonia should be considered when employing this approach. However, the utility of extensive mediastinal lymphadenectomy for this type of tumor remains controversial.

## Supplementary Information

Below is the link to the electronic supplementary material.

Supplementary Material 1

## Data Availability

All the data used in the study are available from the first and corresponding author on reasonable request.

## Abbreviations

EGJC: Esophagogastric junction cancer
TME: Transmediastinal esophagectomy
OS: Overall survival
DFS: Disease-free survival
AC: Adenocarcinoma
SCC: Squamous cell carcinoma
NAC: Neoadjuvant chemotherapy
DCF: Docetaxel+CDDP + 5-FU
LN: Lymph node
C–D: Clavien–Dindo
RLNP: Recurrent laryngeal nerve palsy
CI: Confidence intervals
