# Supplementary material for: Outcomes of transmediastinal esophagectomy for patients with esophagogastric junction cancers
Source: Int J Clin Oncol. 2026 May 27;31(8):1587–96. doi: 10.1007/s10147-026-03068-1 (PMC13401529; doi:10.1007/s10147-026-03068-1)
Supplement: Supplementary file 1 — Supplementary Material 1 [file 10147_2026_3068_MOESM1_ESM.pptx]

## Slide 1
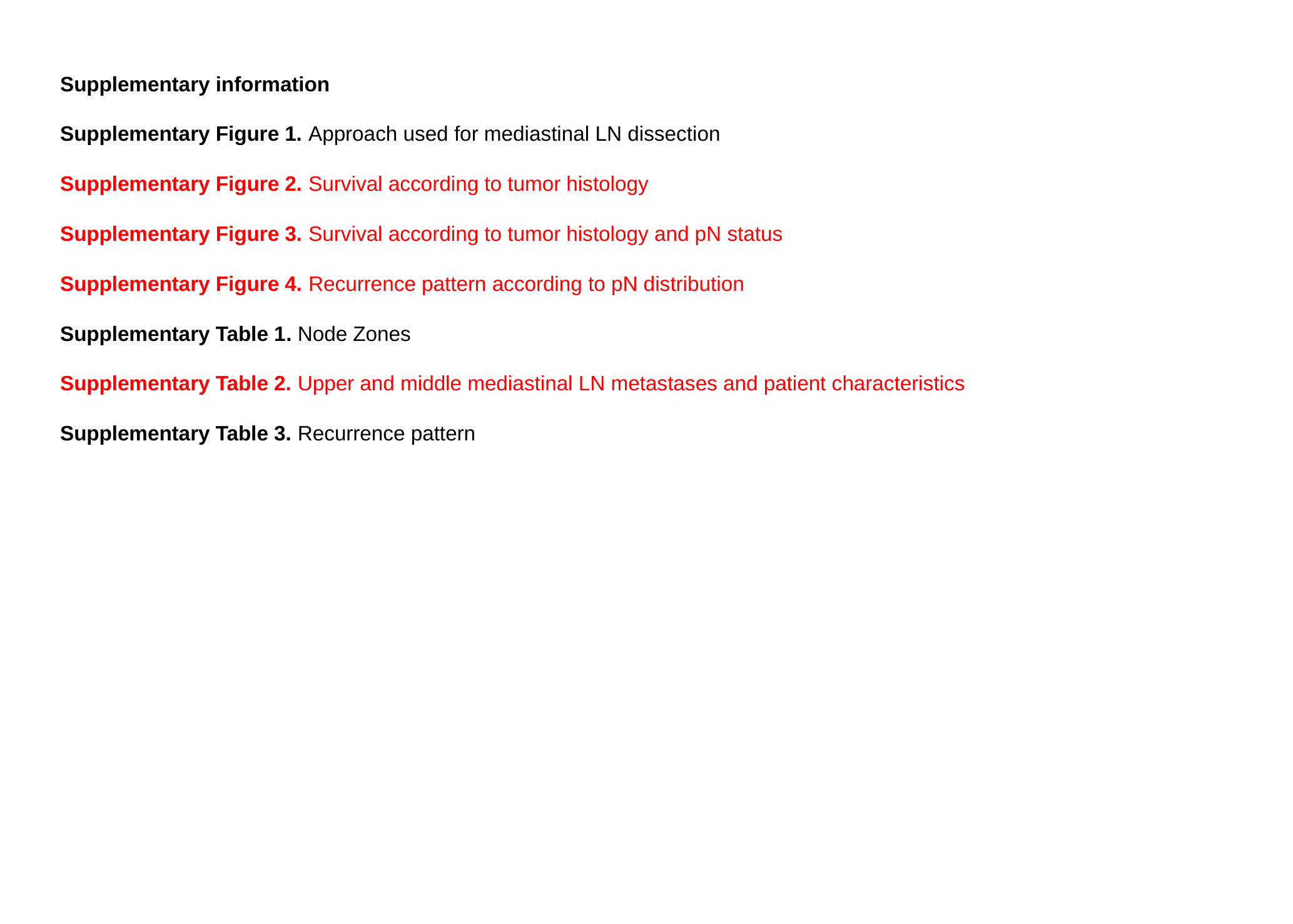

Supplementary information
Supplementary Figure 1. Approach used for mediastinal LN dissection
Supplementary Figure 2. Survival according to tumor histology
Supplementary Figure 3. Survival according to tumor histology and pN status
Supplementary Figure 4. Recurrence pattern according to pN distribution
Supplementary Table 1. Node Zones
Supplementary Table 2. Upper and middle mediastinal LN metastases and patient characteristics
Supplementary Table 3. Recurrence pattern

## Slide 2
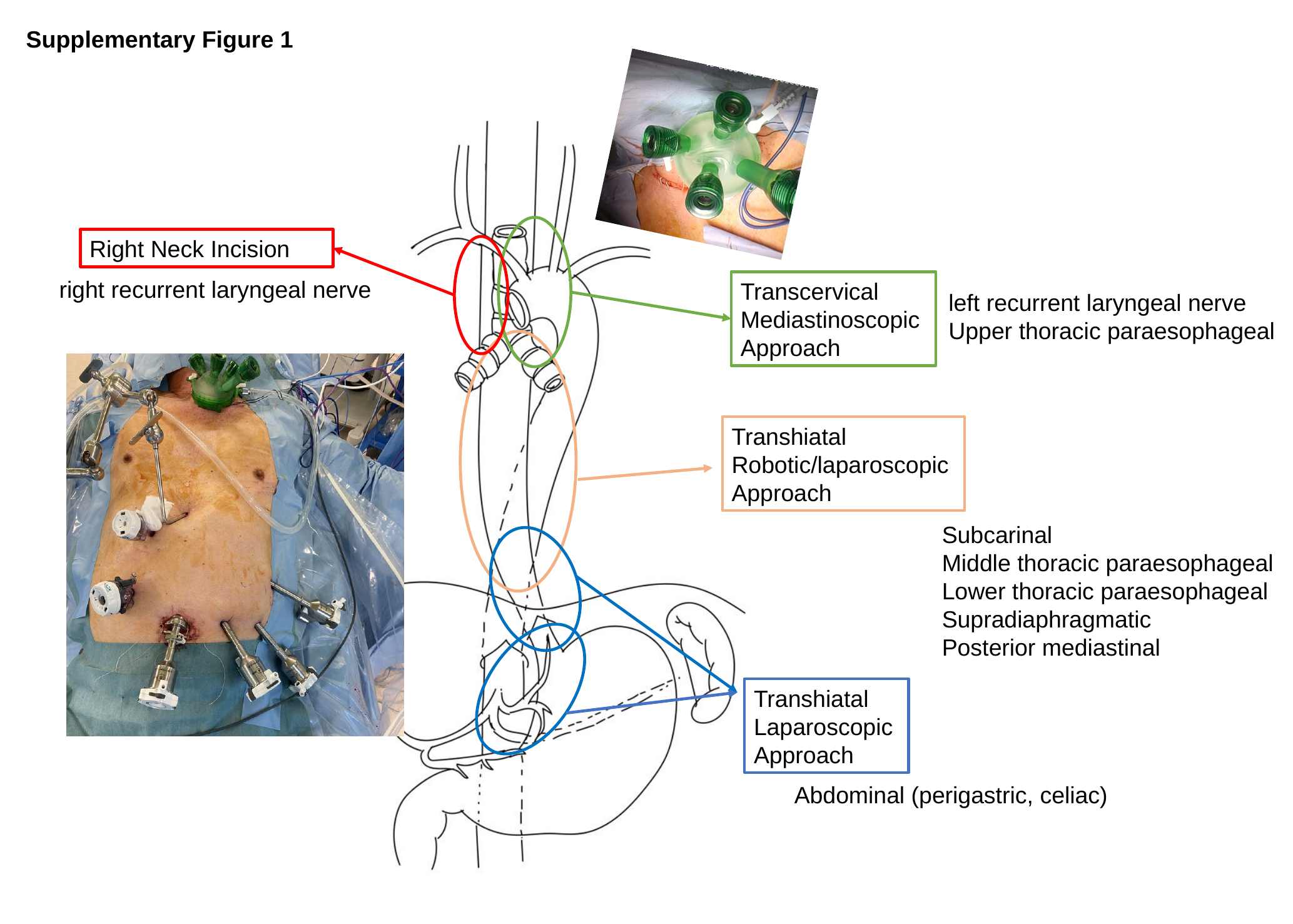

Supplementary Figure 1
Right Neck Incision
right recurrent laryngeal nerve
Transcervical
Mediastinoscopic
Approach
left recurrent laryngeal nerve
Upper thoracic paraesophageal
Transhiatal
Robotic/laparoscopic
Approach
Subcarinal
Middle thoracic paraesophageal
Lower thoracic paraesophageal
Supradiaphragmatic
Posterior mediastinal
Transhiatal
Laparoscopic
Approach
Abdominal (perigastric, celiac)

## Slide 3
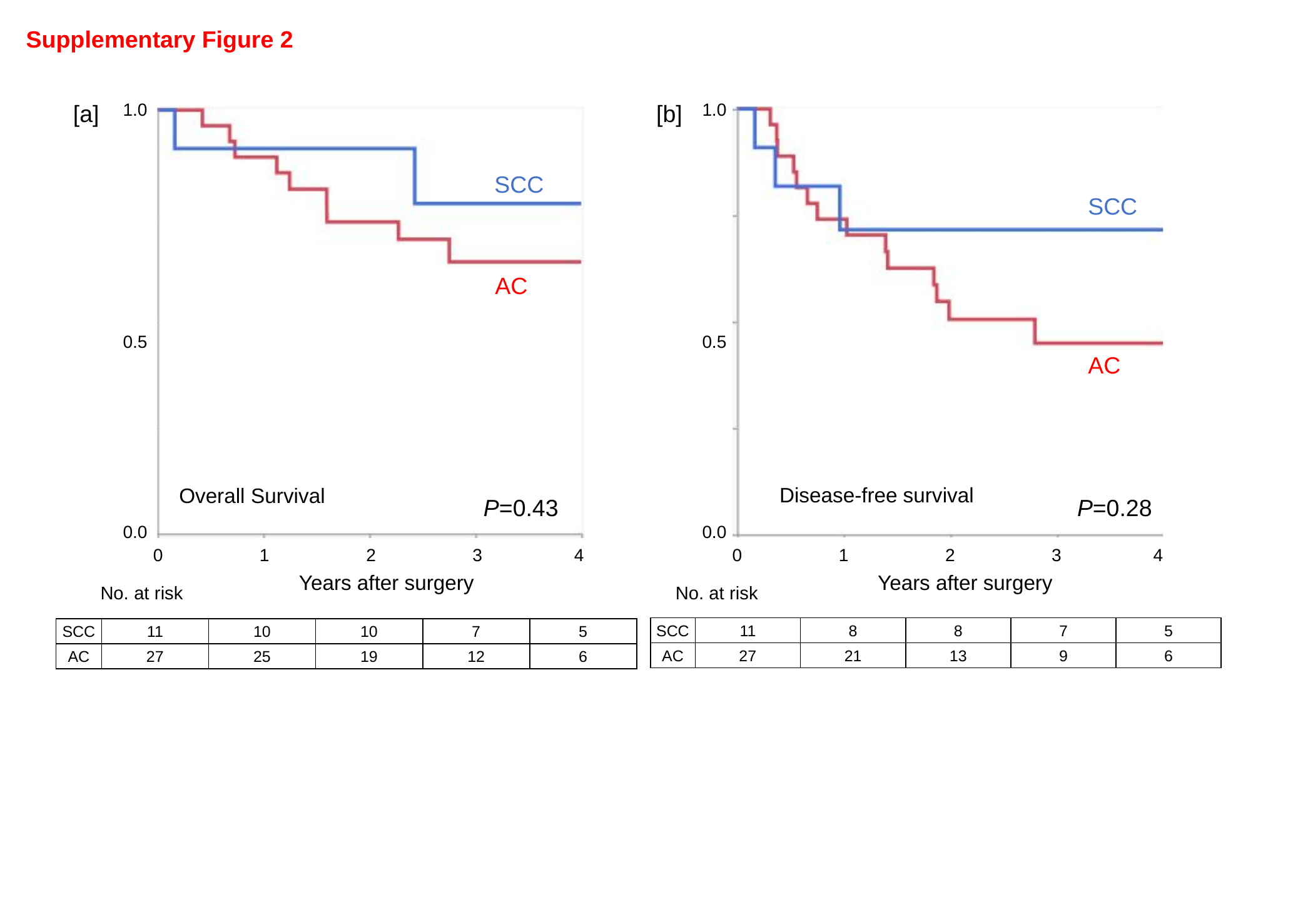

Supplementary Figure 2
[a]
1.0
0.5
0.0
1.0
0.5
0.0
[b]
SCC
SCC
AC
AC
Disease-free survival
Overall Survival
0 1 2 3 4
Years after surgery
P=0.43
P=0.28
0 1 2 3 4
Years after surgery
No. at risk
No. at risk
| SCC | 11 | 8 | 8 | 7 | 5 |
| --- | --- | --- | --- | --- | --- |
| AC | 27 | 21 | 13 | 9 | 6 |
| SCC | 11 | 10 | 10 | 7 | 5 |
| --- | --- | --- | --- | --- | --- |
| AC | 27 | 25 | 19 | 12 | 6 |

## Slide 4
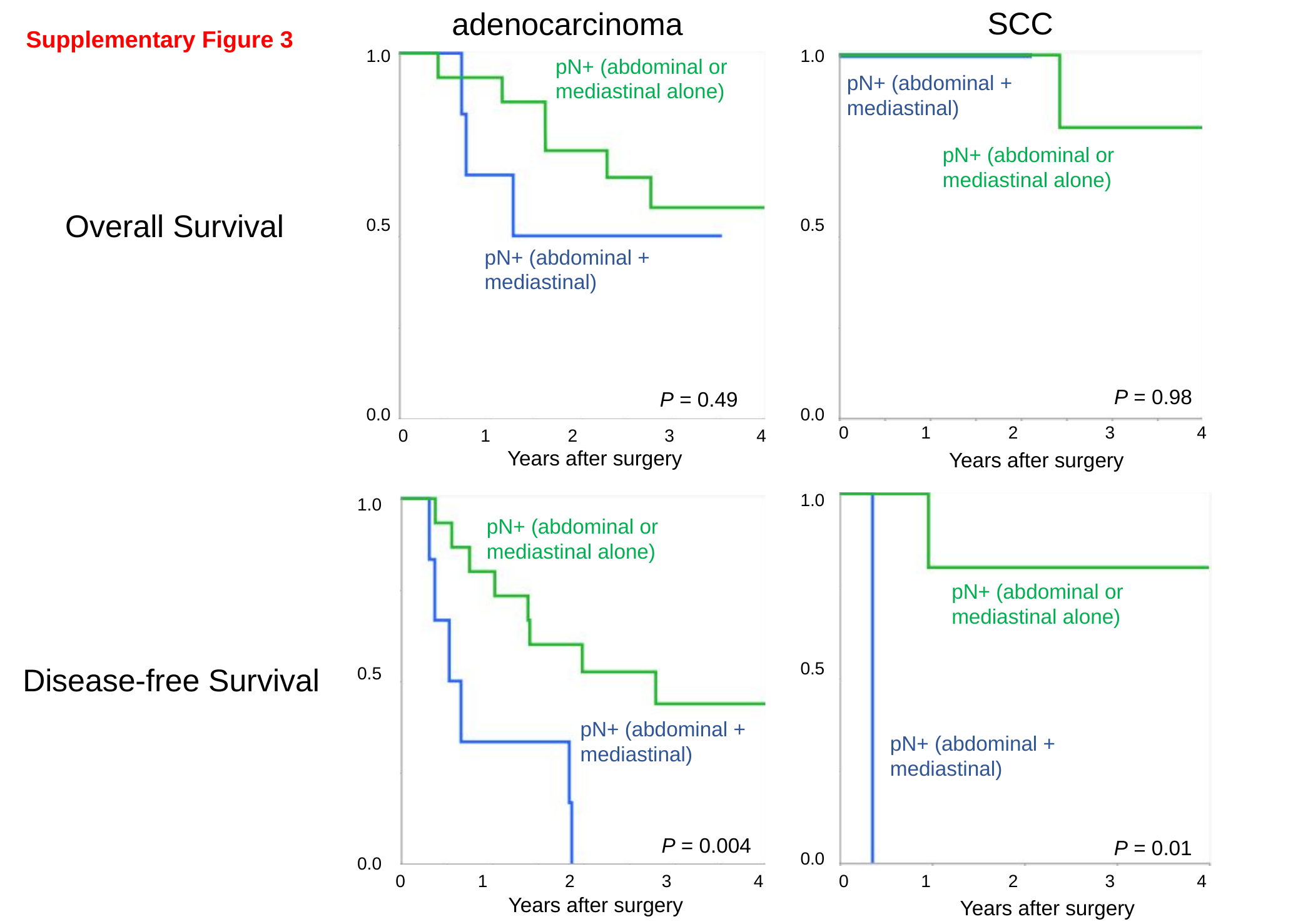

SCC
adenocarcinoma
Supplementary Figure 3
1.0
0.5
0.0
pN+ (abdominal + mediastinal)
pN+ (abdominal or mediastinal alone)
P = 0.98
Years after surgery
0 1 2 3 4
1.0
0.5
0.0
pN+ (abdominal or mediastinal alone)
pN+ (abdominal + mediastinal)
P = 0.49
Years after surgery
0 1 2 3 4
Overall Survival
1.0
0.5
0.0
pN+ (abdominal or mediastinal alone)
pN+ (abdominal + mediastinal)
P = 0.01
Years after surgery
0 1 2 3 4
1.0
0.5
0.0
pN+ (abdominal or mediastinal alone)
pN+ (abdominal + mediastinal)
P = 0.004
Years after surgery
0 1 2 3 4
Disease-free Survival

## Slide 5
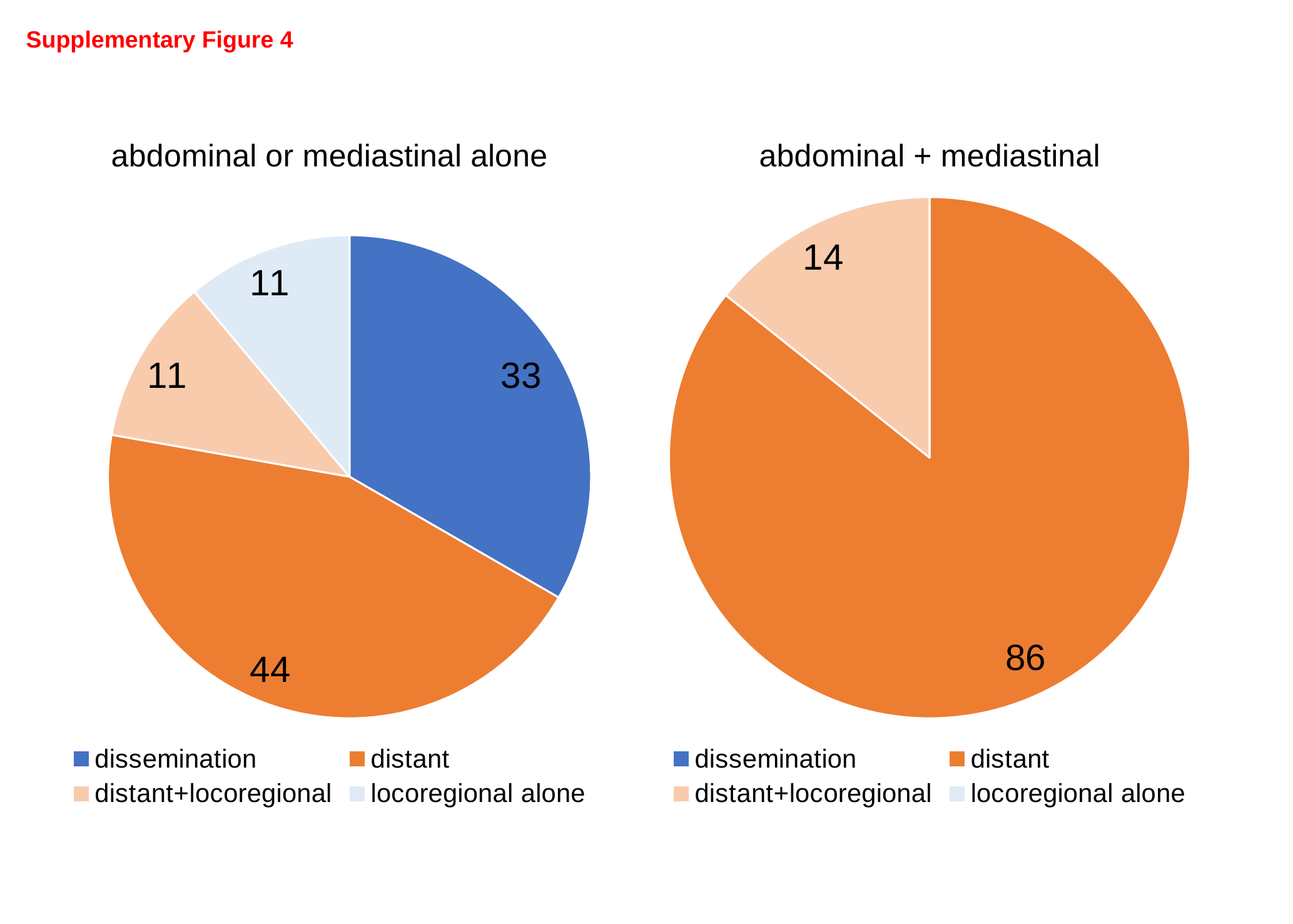

Supplementary Figure 4
### Chart:
| Category | abdominal or mediastinal alone |
|---|---|
| dissemination | 33.333333333333336 |
| distant | 44.44444444444444 |
| distant+locoregional | 11.11111111111111 |
| locoregional alone | 11.11111111111111 |
### Chart:
| Category | abdominal + mediastinal |
|---|---|
| dissemination | 0.0 |
| distant | 85.71428571428571 |
| distant+locoregional | 14.285714285714286 |
| locoregional alone | 0.0 |

## Slide 6
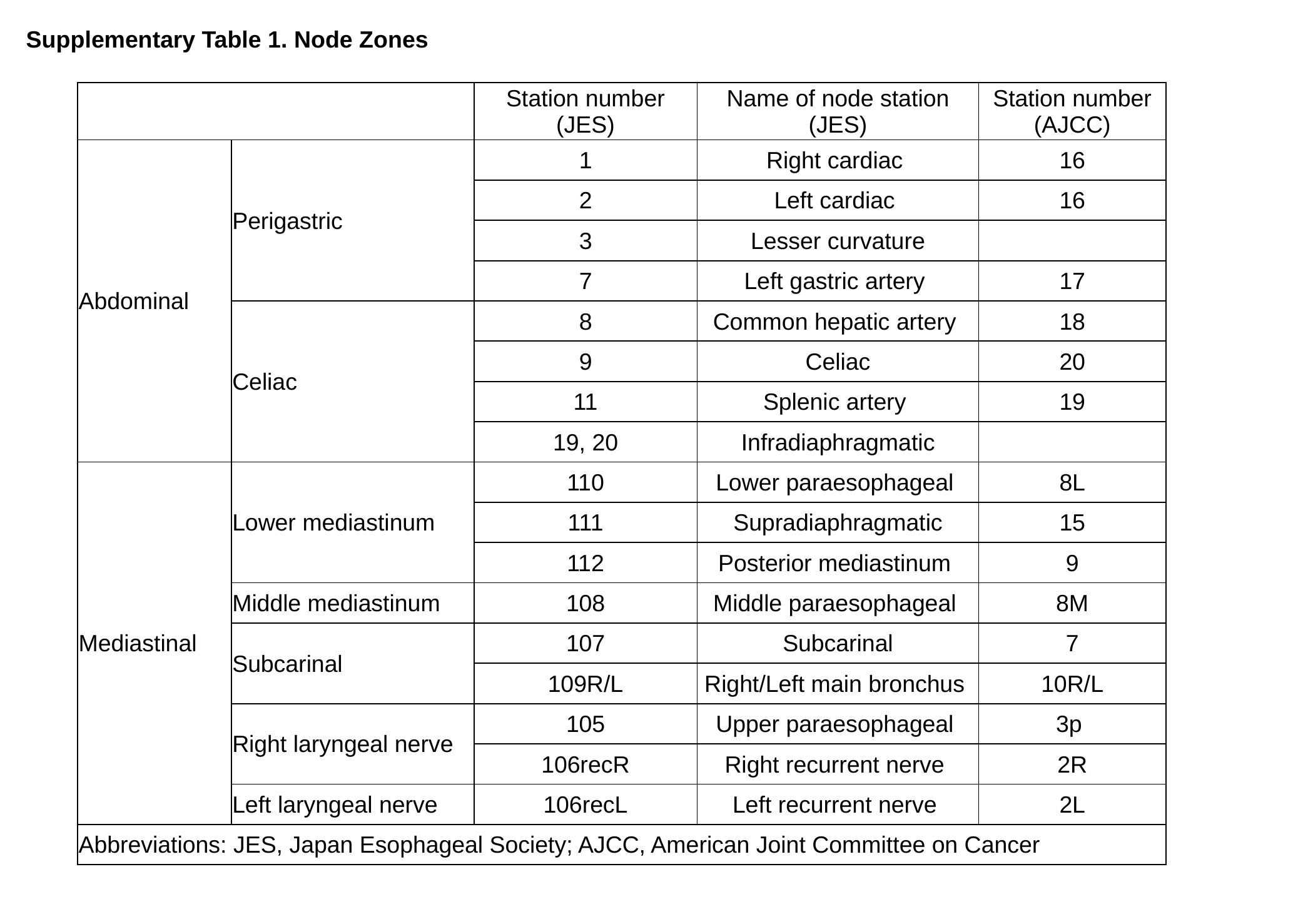

Supplementary Table 1. Node Zones
| | | Station number (JES) | Name of node station (JES) | Station number (AJCC) |
| --- | --- | --- | --- | --- |
| Abdominal | Perigastric | 1 | Right cardiac | 16 |
| | | 2 | Left cardiac | 16 |
| | | 3 | Lesser curvature | |
| | | 7 | Left gastric artery | 17 |
| | Celiac | 8 | Common hepatic artery | 18 |
| | | 9 | Celiac | 20 |
| | | 11 | Splenic artery | 19 |
| | | 19, 20 | Infradiaphragmatic | |
| Mediastinal | Lower mediastinum | 110 | Lower paraesophageal | 8L |
| | | 111 | Supradiaphragmatic | 15 |
| | | 112 | Posterior mediastinum | 9 |
| | Middle mediastinum | 108 | Middle paraesophageal | 8M |
| | Subcarinal | 107 | Subcarinal | 7 |
| | | 109R/L | Right/Left main bronchus | 10R/L |
| | Right laryngeal nerve | 105 | Upper paraesophageal | 3p |
| | | 106recR | Right recurrent nerve | 2R |
| | Left laryngeal nerve | 106recL | Left recurrent nerve | 2L |
| Abbreviations: JES, Japan Esophageal Society; AJCC, American Joint Committee on Cancer | | | | |

## Slide 7
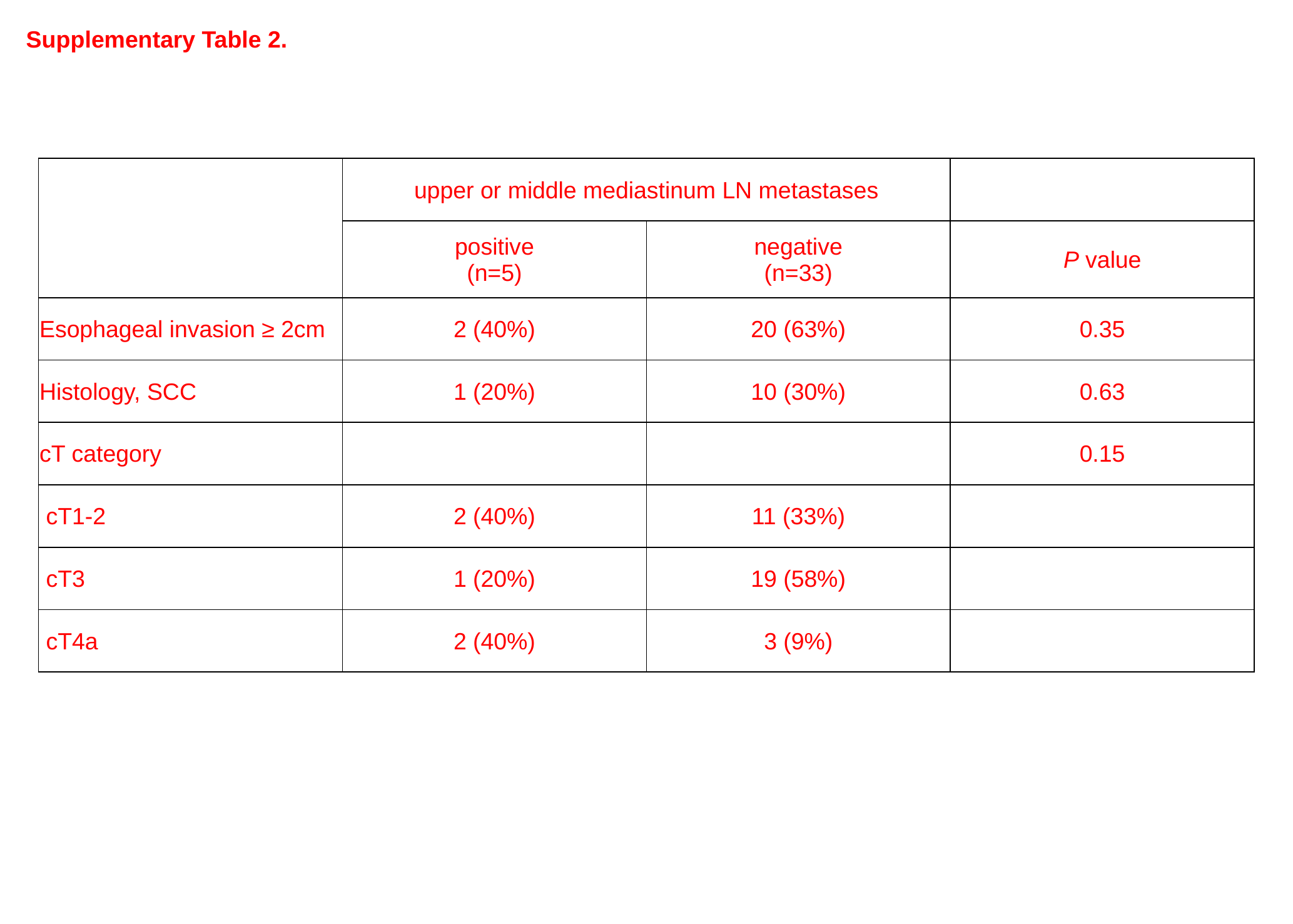

Supplementary Table 2.
| | upper or middle mediastinum LN metastases | | |
| --- | --- | --- | --- |
| | positive (n=5) | negative (n=33) | P value |
| Esophageal invasion ≥ 2cm | 2 (40%) | 20 (63%) | 0.35 |
| Histology, SCC | 1 (20%) | 10 (30%) | 0.63 |
| cT category | | | 0.15 |
| cT1-2 | 2 (40%) | 11 (33%) | |
| cT3 | 1 (20%) | 19 (58%) | |
| cT4a | 2 (40%) | 3 (9%) | |

## Slide 8
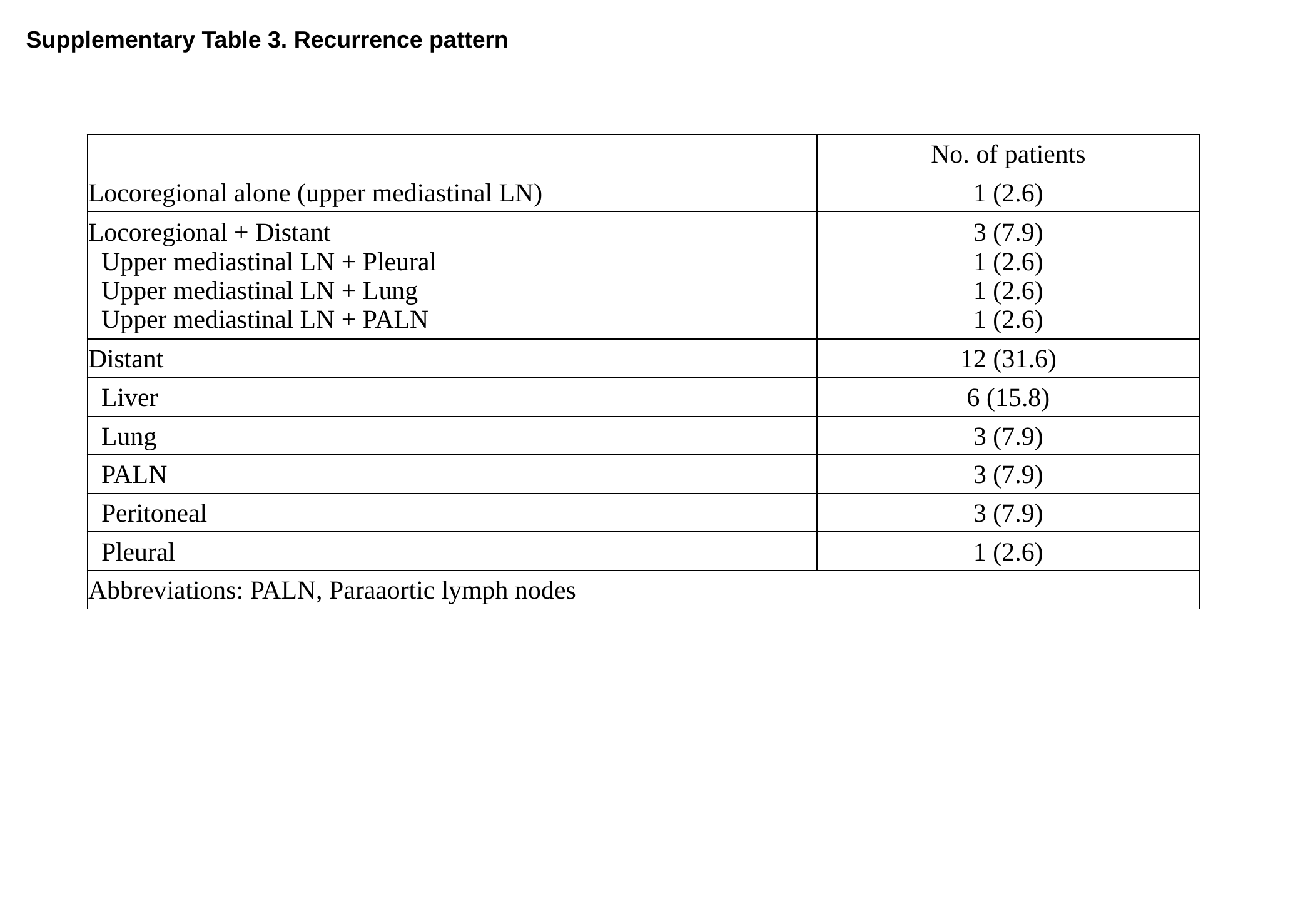

Supplementary Table 3. Recurrence pattern
| | No. of patients |
| --- | --- |
| Locoregional alone (upper mediastinal LN) | 1 (2.6) |
| Locoregional + Distant Upper mediastinal LN + Pleural Upper mediastinal LN + Lung Upper mediastinal LN + PALN | 3 (7.9) 1 (2.6) 1 (2.6) 1 (2.6) |
| Distant | 12 (31.6) |
| Liver | 6 (15.8) |
| Lung | 3 (7.9) |
| PALN | 3 (7.9) |
| Peritoneal | 3 (7.9) |
| Pleural | 1 (2.6) |
| Abbreviations: PALN, Paraaortic lymph nodes | |
